# Cardiac Patients’ Walking Activity Determined by a Step Counter in Cardiac Telerehabilitation: Data From the Intervention Arm of a Randomized Controlled Trial

**DOI:** 10.2196/jmir.5191

**Published:** 2016-04-04

**Authors:** Charlotte Thorup, John Hansen, Mette Grønkjær, Jan Jesper Andreasen, Gitte Nielsen, Erik Elgaard Sørensen, Birthe Irene Dinesen

**Affiliations:** ^1^ Department of Cardiothoracic Surgery Aalborg University Hospital Aalborg Denmark; ^2^ Department of Clinical Medicine Aalborg University Aalborg Denmark; ^3^ Laboratory for Cardio technology, Medical Informatics Group Department of Health Science and Technology, Faculty of Medicine Aalborg University Aalborg Denmark; ^4^ Clinical Nursing Research Unit Aalborg University Hospital Aalborg Denmark; ^5^ Department of Cardiology Vendsyssel Hospital Hjoerring Denmark; ^6^ Laboratory of Assistive Technologies - Telehealth and Telerehabilitation, SMI Department of Health Science and Technology, Faculty of Medicine Aalborg University Aalborg Denmark

**Keywords:** heart disease, rehabilitation, step counters, physical activity, telerehabilitation

## Abstract

**Background:**

Walking represents a large part of daily physical activity. It reduces both overall and cardiovascular diseases and mortality and is suitable for cardiac patients. A step counter measures walking activity and might be a motivational tool to increase and maintain physical activity. There is a lack of knowledge about both cardiac patients’ adherence to step counter use in a cardiac telerehabilitation program and how many steps cardiac patients walk up to 1 year after a cardiac event.

**Objective:**

The purpose of this substudy was to explore cardiac patients’ walking activity. The walking activity was analyzed in relation to duration of pedometer use to determine correlations between walking activity, demographics, and medical and rehabilitation data.

**Methods:**

A total of 64 patients from a randomized controlled telerehabilitation trial (Teledi@log) from Aalborg University Hospital and Hjoerring Hospital, Denmark, from December 2012 to March 2014 were included in this study. Inclusion criteria were patients hospitalized with acute coronary syndrome, heart failure, and coronary artery bypass grafting or valve surgery. In Teledi@log, the patients received telerehabilitation technology and selected one of three telerehabilitation settings: a call center, a community health care center, or a hospital. Monitoring of steps continued for 12 months and a step counter (Fitbit Zip) was used to monitor daily steps.

**Results:**

Cardiac patients walked a mean 5899 (SD 3274) steps per day, increasing from mean 5191 (SD 3198) steps per day in the first week to mean 7890 (SD 2629) steps per day after 1 year. Adherence to step counter use lasted for a mean 160 (SD 100) days. The patients who walked significantly more were younger (*P*=.01) and continued to use the pedometer for a longer period (*P*=.04). Furthermore, less physically active patients weighed more. There were no significant differences in mean steps per day for patients in the three rehabilitation settings or in the disease groups.

**Conclusions:**

This study indicates that cardiac telerehabilitation at a call center can support walking activity just as effectively as telerehabilitation at either a hospital or a health care center. In this study, the patients tended to walk fewer steps per day than cardiac patients in comparable studies, but our study may represent a more realistic picture of walking activity due to the continuation of step counter use. Qualitative studies on patients’ behavior and motivation regarding step counter use are needed to shed light on adherence to and motivation to use step counters.

**Trial Registration:**

ClinicalTrails.gov NCT01752192; https://clinicaltrials.gov/ct2/show/NCT01752192 (Archived by WebCite at http://www.webcitation.org/6fgigfUyV)

## Introduction

Cardiac diseases are the main causes of death and account for 13% to 15% of all deaths worldwide [1] and 24.8% of all deaths in Europe [2]. Cardiac rehabilitation aims to improve cardiac patients’ functional capacity, recovery, psychosocial well-being, and health-related quality of life through a multidisciplinary intervention consisting of physical activity support, exercise training, diet and weight counseling, psychosocial coping, and management of the disease [2]. Cardiac rehabilitation is vital for recovery after cardiac disease, yet cardiac rehabilitation has poor compliance and adherence [2,3]. Home-based cardiac rehabilitation programs, such as cardiac telerehabilitation, have been introduced to increase access, participation, and adherence. Cardiac telerehabilitation is defined as cardiac rehabilitation that uses information and communication technology to improve health and lifestyle by monitoring and communicating through interactive tools. Cardiac telerehabilitation has proven to be just as effective in decreasing morbidity and mortality as center- and hospital-based cardiac rehabilitation programs [4-6]. Furthermore, cardiac telerehabilitation has the potential to reach citizens who live long distances from rehabilitation centers [4-12]. Physical activity decreases cardiovascular mortality and morbidity [13-20]. Walking is a simple physical activity that forms a large part of daily physical activity in both sedentary and active individuals, reducing both overall and cardiovascular disease mortality [21]. As a result, walking strategies need to be included in cardiac rehabilitation. Walking is suitable for cardiac patients because it is safe and feasible for almost all patients [22-24]. Step counters are recognized tools to count steps and measure walking activity, and they represent an important motivational tool to measure and increase adherence to physical activity [17,25,26]. Studies of cardiac patients’ use of step counters show an increase of physical activity with their use [18,21,25,27-31]. Cardiac patients who receive walking interventions have higher levels of walking activity compared to control groups, and their walking activity increases over 1 year [16-18].

People are considered physically active when they perform more than 30 minutes of moderate to intense activity per day (on most days of the week) [32]. Researchers agree that approximately 7000-10,000 steps per day is equivalent to 30 minutes per day of moderate to intense physical activity. More than 10,000 steps per day is considered highly active [15,17,24,32]. There is a lack of knowledge about cardiac patients’ adherence to, and use of, step counters during rehabilitation. We also lack knowledge regarding how many steps cardiac patients walk during the period up to 1 year after a cardiac event [16,17]. A 1-year follow-up is considered relevant because sustained behavioral changes require a long observation period [33]. In an attempt to increase patients’ physical activity, it is important to identify realistic and appropriate goals [21,29,31,34]. Six studies have been identified that explore the amount of walking activity by patients with cardiac disease [15-18,21,35] and these studies show diversity in results. Further studies on cardiac patients’ walking activity and use of step counters are needed.

This study is part of a larger Danish research project, Teledi@log, in which a cardiac telerehabilitation program has been developed and tested as a randomized controlled trial for patients with heart diseases. This paper focuses explicitly on cardiac patients’ walking activity. The walking activity will be analyzed in relation to the duration of step counter use to determine correlations between walking activity, demographics, and medical and rehabilitation data.

## Methods

### Cardiac Telerehabilitation

The patients selected were participants from a randomized controlled cardiac telerehabilitation study, Teledi@log (ClinicalTrails.gov NCT01752192), and were included from December 2012 to March 2014. The Teledi@log project was approved by the Danish Ethical Committee (N-20120051). The general objective of Teledi@log was to tailor cardiac telerehabilitation based on the patient’s individual needs. The telerehabilitation program lasted for 3 months. Patients in the intervention group were provided with a step counter, a scale, a sphygmomanometer, and a tablet. The tablet contained a tailored personal health record (PHR) for health information and communication between the patient and health professionals. The patients measured blood pressure, pulse, and weight twice a week and number of steps recorded daily on a step counter. Data were transmitted wirelessly from the devices to the PHR. Based on the patient’s individual condition, the rehabilitation nurse created a tailored rehabilitation plan for each patient, containing an activity plan with goals for daily steps. This was done in accordance with European Association of Cardiovascular Prevention and Rehabilitation recommendations [3] and in collaboration with the patient before discharge from the hospital. The plan was displayed in the PHR. Both the patient and the health professionals at the hospital and health care center had access to and communicated via the patient’s PHR. All patients had personal goals for daily steps in the PHR. In addition to access to health information, the step counter was the only telerehabilitation technology that the patients retained after 3 months, allowing them to continue monitoring steps for 12 months. The telerehabilitation technology provided the patients with insights into their own walking activity, enabling them to monitor and tailor their own activity plans. All patients were assigned a personal nurse attached to either the health care center or the hospital. The control group received traditional cardiac rehabilitation. In Denmark, Danish national guidelines specify that cardiac patients can be offered either cardiac rehabilitation at a hospital or at a health care center [36]. To match this in the intervention group, cardiac patients in the Teledi@log trial selected one of three rehabilitation settings: call center and telerehabilitation, individualized cardiac telerehabilitation at a community health care center, or individualized cardiac telerehabilitation at the hospital (Textbox 1).

**Settings for cardiac telerehabilitation.**

1. Call center (contact person: cardiac nurse)

- A cardiac nurse from the hospital was in charge of the patient’s rehabilitation and all rehabilitation activities were provided through the personal health record and in collaboration with the rehabilitation nurse in accordance with European Association of Cardiovascular Prevention and Rehabilitation, individualized activities were planned (patient decided what activities to follow)
- Follow-up time was based on individual needs
- Self-monitoring
- Step counter

2. Health care center (contact person: cardiac rehabilitation nurse)

- A rehabilitation nurse from the health care center was in charge of the patient’s rehabilitation and all rehabilitation activities were provided through the personal health record and in collaboration with the rehabilitation nurse in accordance with recommendations from European Association of Cardiovascular Prevention and Rehabilitation; furthermore, the rehabilitation consisted of individual and group sessions once or twice a week for 12 weeks
- Group consultation and exercise sessions took place together with other cardiac patients
- Follow-up time was based on individual needs
- Group exercise
- Self-monitoring
- Step counter

3. Hospital (contact person: cardiac nurse)

- A cardiac nurse from the hospital was in charge of the patient’s rehabilitation and all rehabilitation activities were provided through the personal health record and in collaboration with the rehabilitation nurse, in accordance with recommendations from European Association of Cardiovascular Prevention and Rehabilitation; furthermore, the rehabilitation consisted of individual and group sessions once or twice a week for 12 weeks
- Consultation and exercise sessions took place in a group with other cardiac patients
- Follow-up time was based on individual needs
- Group exercise
- Self-monitoring
- Step counter

### Participants and Recruitment

**Figure 1:**
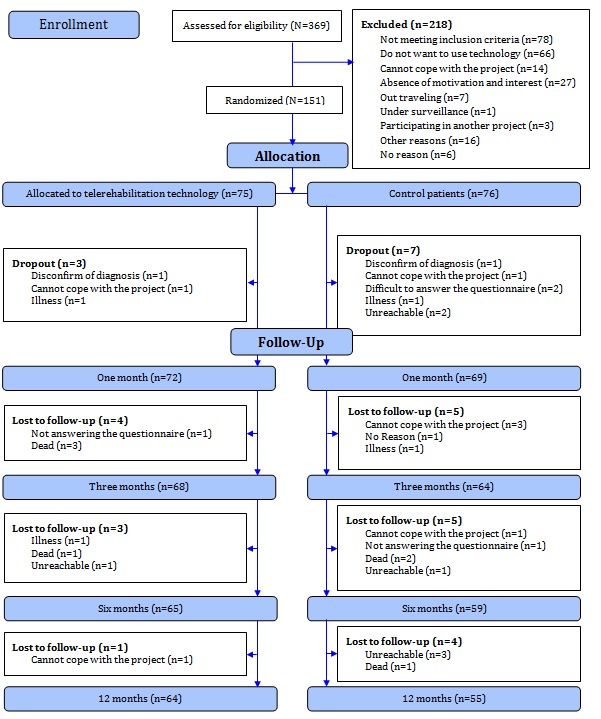
Teledi@log CONSORT flow diagram.

A computer-based block randomization in groups of 10 was performed. In total, 151 cardiac patients participated in Teledi@log. Of these, 72 were in the intervention group who received telerehabilitation technologies, including the step counter. Eight of the patients in the intervention group dropped out during the 1-year study period (four died, one had severe progression of illness, one was unreachable at follow-up, and two could not cope with or finish the project), leaving 64 patients for this step counter substudy. Figure 1 shows the CONSORT flow diagram of Teledi@log, with the 64 patients in this study shown as the intervention group.

Patients were recruited from Aalborg University Hospital, Aalborg, Denmark, and Vendsyssel Hospital, Hjoerring, Denmark. The inclusion criteria were patients hospitalized with acute coronary syndrome (ACS), heart failure (ejection fraction <40%), coronary artery bypass graft (CABG), or valve replacement/mitral valve repair. Patients were excluded in cases of pregnancy or breastfeeding, or if they did not speak Danish. Telerehabilitation nurses at the participating hospitals reviewed patients’ charts for eligibility and eligible patients were approached. Those who agreed to participate signed an informed consent form. Demographic data were registered by the nurse. Furthermore, the patients were instructed in how to use the telerehabilitation technologies and measurement started immediately after discharge. Fourteen days after inclusion, each patient was visited by a research assistant to ensure that the patients were using the telerehabilitation technologies correctly.

**Figure 2:**
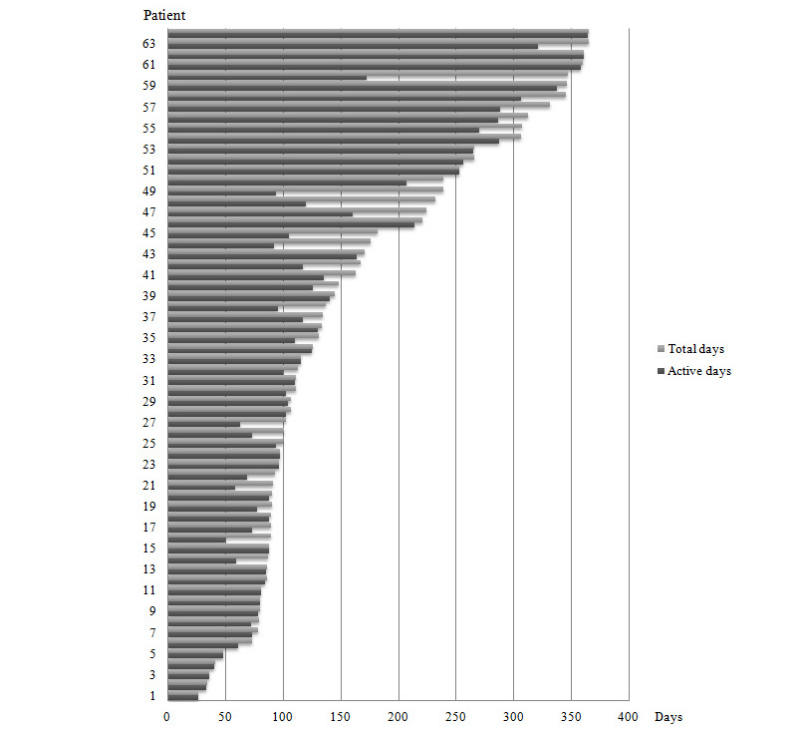
Duration of Fitbit use. Total days (gray) in relation to active days (black) of use for each patient.

### Step Measurements

The number of steps was assessed using the Fitbit Zip step counter (Fitbit Inc, San Francisco, CA, USA) [37].

Patients were asked to fasten the Fitbit at the breast pocket or hip during all waking hours (except for bathing and swimming) for at least 3 months after hospital discharge and for a period of up to 1 year. Before being used, the Fitbit was programmed with the patient’s date of birth, gender, weight, and height to ensure accuracy of the step counts obtained. Fitbit Zip uses a 3-axis accelerometer and converts accelerometer data into step data. Step data were continually visible on the Fitbit’s display; each day at midnight, the steps per day were downloaded to the patient’s PHR. For statistical analysis, each patient’s step data were downloaded on a secure database at intervals of 1 minute for 365 days from the day of inclusion. Bäck et al [35] proposed a graduated step index to describe activity in cardiac patients: (1) <3000 steps per day (low activity/sedentary), (2) 3000-9999 steps per day (medium activity), and (3) ≥10,000 steps per day (high activity). This classification was used in the presentation of data.

### Statistical Analysis

Means with standard deviation are presented for continuous variables and proportions (%) for categorical variables. Mean body mass index (BMI) was measured as the difference between baseline BMI and BMI at 3 months divided by 2.

The grand mean for every patient was measured as the mean of all the patients’ active days. *Active days* were defined as days with more than 100 steps per day. The low cut-off point of ≥100 steps per day was set to capture very low activity and still avoid failure measurement when the Fitbit was moved around and not worn. Activity of less than 100 steps per day was considered to indicate moving the Fitbit around but not wearing it. Furthermore, steps per day was also measured as a weekly mean at day 7 and at 1 month, 3 months, 6 months, 9 months, and 12 months. Patients who stopped using the Fitbit were called “nonusers” from the time they stopped.

Duration of use was measured as total days*,* counted from the starting day to the final day of Fitbit use. The final day was determined as the last day of ≥4 consecutive active days of Fitbit recordings despite any temporary break in use. If the patients had less than four active days of consecutive Fitbit recordings, the use was considered terminated.

The association between Fitbit groups and baseline characteristics was tested using 1-way ANOVA for continuous data values and the Fisher exact test for categorical data. In the case of significant difference, a post hoc Bonferroni test was carried out. For nonnormally distributed continuous data, a Kruskal-Wallis test was done (days and total days) and a *t* test was performed for gender-sorted grand mean of steps walked. To test association between mean numbers of steps walked and termination of step counter use, a repeated measure logistic regression analysis was carried out. All tests were considered statistically significant if *P*<.05. MATLAB release 2014b (MathWorks, Natick, MA, USA) and STATA version 13.1 (StataCorp, College Station, TX, USA) were used for statistical analyses.

## Results

### Patient Characteristics

Patient baseline characteristics are shown in Table 1. Of the 64 patients in our sample, 14 (22%) were classified as low active with a mean of <3000 steps per day and 14% (9/64) were highly active, walking ≥10,000 steps per day. The remaining 41 patients (64%) were medium active, walking between 3000 and 9999 steps per day. The mean age of the entire sample was 62.8 years (range 35-88 years). There was a significant difference in age between the activity groups (*P*=.01). Patients in the low activity group were significantly older than patients in both the medium activity group (*P*=.03) and the high activity group (*P*=.02). The mean ages in the three activity groups of low, medium, and high were 70.7 (SD 10.7), 61.1 (SD 11.4), and 58.2 (SD 8.3) years, respectively. Males represented 51 of 64 (80%) participants. Even though the less active patients’ mean BMI was higher, this was not significant. Almost half (48%, 31/64) of the patients had a primary diagnosis of ACS and 21 of 64 patients (33%) were treated with surgery (CABG or valve replacement/mitral valve replacement). Eight patients (11%) had heart failure and five patients (8%) had both heart failure and ACS. For cardiac telerehabilitation, 29 of 64 patients (45%) chose the health care center and 23 of 64 (36%) chose the hospital. The remaining 12 patients (19%) chose the call center.

**Table 1:** Patient baseline characteristics.

Characteristic^a^			All patients	Activity level (steps/day)			*P* ^b^
				Low (<2999)	Medium (3000-9999)	High (≥10,000)	
**Demographic variables**							
	Participants, n (%)		64 (100)	14 (22)	41 (64)	9 (14)	
	Age (years), mean (SD)		62.8 (11.5)	70.7 (10.7)	61.1 (11.4)	58.2 (8.3)	.01^c^
	**Sex, n (%)**						.29
		Male	51 (80)	13 (20)	30 (47)	8 (13)	
		Female	13 (20)	1 (2)	11 (17)	1 (2)	
	BMI (kg/m^2^), mean (SD)		28 (5.1)	29.7 (5.1)	27.7 (5.4)	27.0 (4.2)	.38
**Primary diagnosis or treatment, n (%)**							
	ACS		33 (48)	6 (9)	20 (31)	7 (11)	
	Surgery^d^		18 (33)	6 (9)	12 (19)	0 (0)	
	Heart failure		8 (11)	1 (2)	5 (8)	2 (3)	
	ACS & heart failure		5 (8)	1 (2)	4 (6)	0 (0)	
**Cardiac telerehabilitation, n (%)**							.08
	Health care center		29 (45)	7 (11)	20 (31)	2 (3)	
	Hospital		23 (36)	3 (5)	17 (27)	3 (5)	
	Call center		12 (19)	4 (6)	4 (6)	4 (6)	

^a^ ACS: acute coronary syndrome; BMI: body mass index.

^b^ *P* value for comparison of all three activity groups (low, medium, and high).

^c^ Post hoc Bonferroni corrected values: low versus medium activity groups (*P*=.03), low versus high activity groups (*P*=.02), and medium versus high activity groups (*P*<.99).

^d^ Surgery includes valve replacement, mitral valve repair, and coronary artery bypass grafting.

### Duration of Step Counter Use

Two patients used the Fitbit for a total of 365 days each; the overall mean total days was 160 (SD 100, range 26-365 days). Patients in the low, medium, and high activity groups used the pedometer for a mean of 109 (SD 56), 168 (SD 103), and 208 (SD 112) days, respectively. There was a significant difference between both the low and high activity groups in total days of Fitbit use (*P=*.01). Active days comprised 139 (SD 93) of 160 (87%) total days. There was a significant difference between both the low and medium activity groups (*P=*.01) and the low and high activity groups (*P=*.003) in active days of Fitbit use (Table 2).

**Table 2:** Duration of step counter use and mean daily steps.

Step counter use			All patients	Activity level (steps/day)			*P*
				Low (<2999)	Medium (3000-9999)	High (≥10,000)	
**Duration of use**							
	Total days, mean (SD)		160 (100)	109 (56)	168 (103)	208 (112)	.04^a,b^
	Active days, mean (SD)		139 (93)	79 (26)	148 (97)	189 (102)	.006^a,c^
	Active days/total days, %		87	72	88	91	
**Walking activity (steps/day), mean (SD)**							
	Grand mean		5899 (3151)	1996 (716)	6016 (1784)	11,439 (440)	
	**Gender**						.82^a^
		Male	5853 (3274)	2064 (696)	6008 (1785)	11,430 (469)	
		Female	6078 (2725)	1105 (0)	6037 (1869)	11,501 (0)	
	**Week**						.004^d^
		Week 1 (7 days)	5191 (3198)	1578 (500)	5366 (2306)	9611 (2995)	
		Week 4 (30 days)	6362 (3834)	1807 (780)	6305 (2536)	12,697 (1678)	
		Week 13 (90 days)	6186 (3013)	2304 (1189)	6073 (2271)	10,637 (1095)	
		Week 26 (180 days)	6794 (3518)	808 (0)	6506 (3569)	9011 (884)	
		Week 39 (270 days)	8235 (4220)	0 (0)	5960 (2461)	12,784 (3127)	
		Week 52 (365 days)	7890 (2629)	0 (0)	7426 (2730)	9050 (2811)	
	**Primary diagnose or treatment**						.12^a^
		ACS^e^	6549 (3149)	2194 (712)	6127 (1558)	11,588 (354)	
		Surgery^f^	4781 (3023)	1879 (653)	6058 (845)	0 (0)	
		Heart failure	7340 (3190)	2636 (0)	5949 (2605)	10,916 (306)	
		ACS & heart failure	4505 (163)	865 (0)	5415 (845)	0 (0)	
	**Cardiac telerehabilitation**						.34^a^
		Health care center	5324 (2579)	2197 (585)	5779 (1243)	11,737 (333)	
		Hospital	6128 (3084)	2082 (980)	5950 ( 2242)	11,187 (518)	
		Call center	6847 (4353)	1578 (751)	7485 (1608)	11,478 (418)	

^a^ *P* value for comparison of all three activity groups (low, medium, and high).

^b^ Post hoc 2-sample Wilcoxon rank-sum test values: low versus medium activity groups (*P*=.05), low versus high activity groups (*P*=.01), and medium versus high activity groups (*P*=.22).

^c^ Post hoc 2-sample Wilcoxon rank-sum values: low versus medium activity groups (*P*=.01), low versus high activity groups (*P*=.003), and medium versus high activity groups (*P*=.21).

^d^ *P* value for correlation between termination of step counter use and mean steps walked at the specified days.

^e^ ACS: acute coronary syndrome.

^f^ Surgery includes valve replacement, mitral valve repair, and coronary artery bypass grafting.

**Figure 3:**
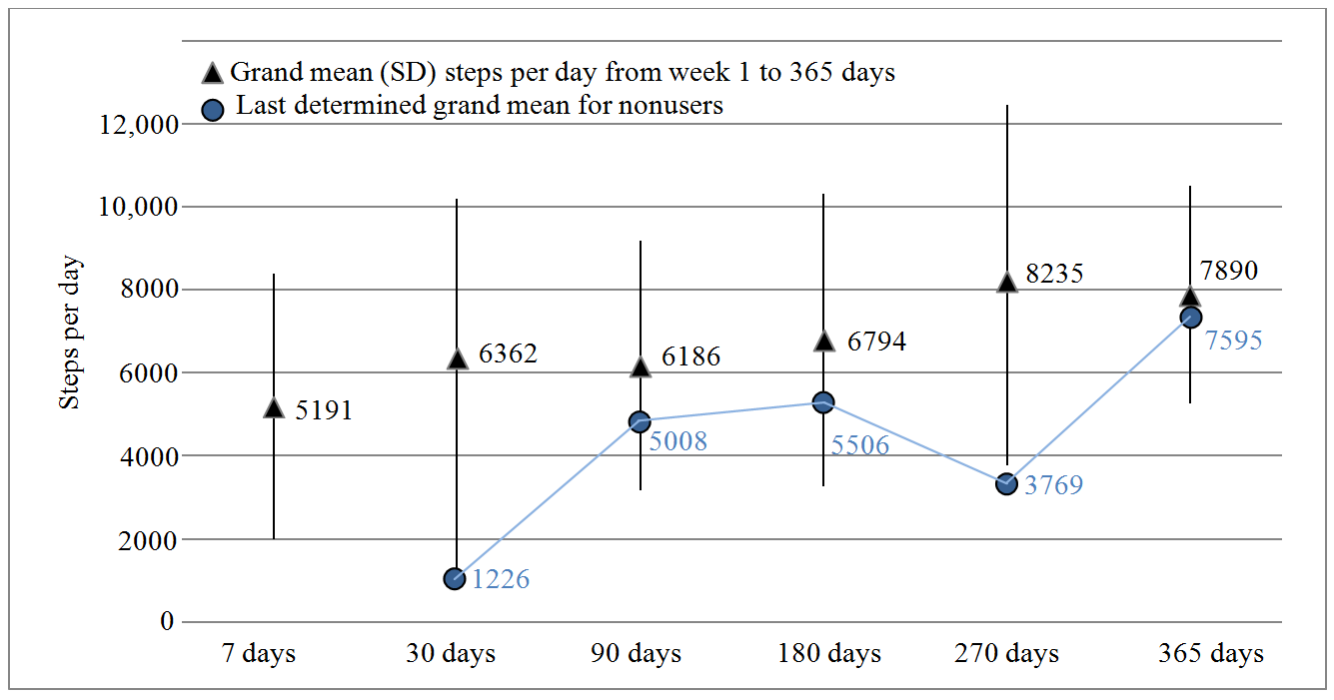
Grand mean steps per day and standard deviation (length of whiskers) at different days for both users and nonusers.

### Step Measurement

The grand mean for all patients for all active days was 5899 (SD 3151) steps per day. The grand mean steps per day were as follows: day 7: mean 5191, SD 3198; day 30: mean 6362, SD 3834; day 90: mean 6186, SD 3013; day 180: mean 6794, SD 3518; day 270: mean 8235, SD 4220; and day 365: mean 7890, SD 2629 steps per day indicating an increase in walking activity over time (Table 2). Knowing that the increase in walking activity could be a result of the low activity patients’ termination of step counter use, the week mean of nonusers (the last determined) was calculated (Figure 3) together with the increase in patients’ grand mean. In addition, a linear regression revealed a significant relationship between termination of step counter use and low week mean steps at different weeks (*P*=.004) (Table 2). Despite the slight increase in nonusers’ weekly means, it cannot be ruled out that the increase in steps per week over the year might be due to the dropping out of those patients with low walking activity.

**Figure 4:**
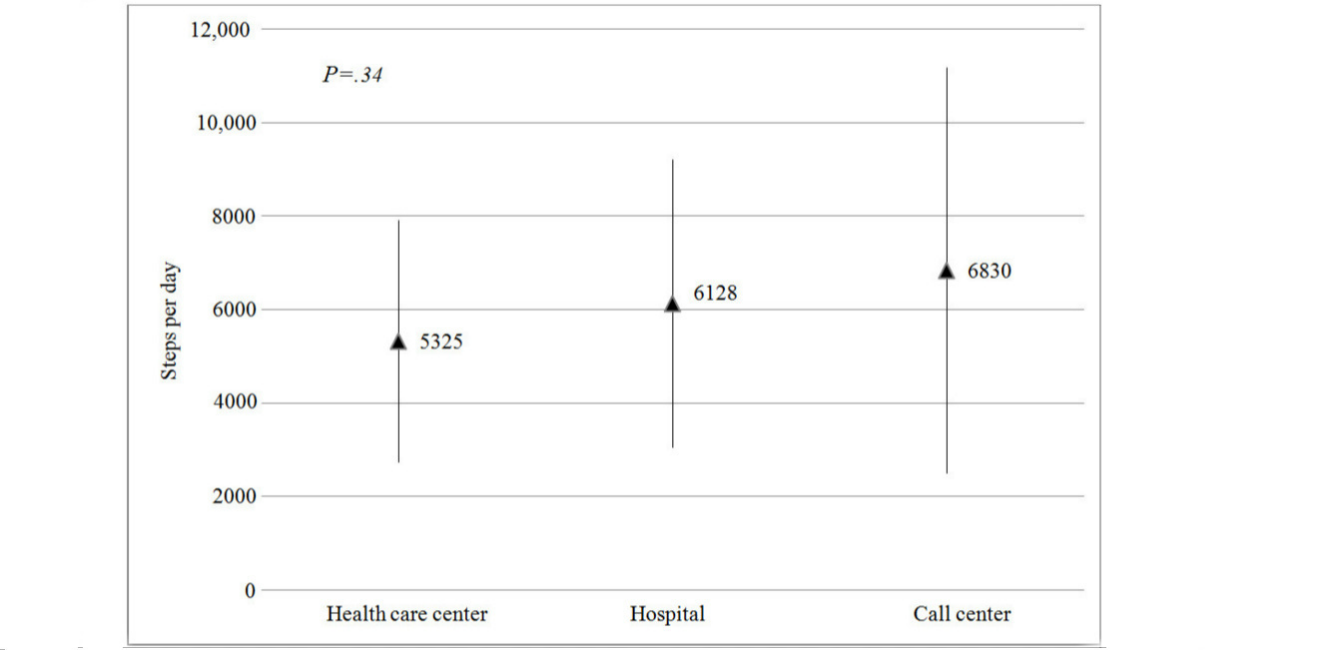
Mean steps per day and standard deviation (length of whiskers) at the three cardiac telerehabilitation settings.

No significant association was found between gender and mean steps per day (*P*=.82) in the different activity groups. There were no significant differences in mean steps per day between the four treatment groups (*P*=.12). Patients who choose the call center for cardiac telerehabilitation had the highest mean steps per day (mean 6847, SD 4353) and patients using the health care center for cardiac telerehabilitation had the lowest (mean 5324, SD 2579 steps/day). There was no significant relation between choice of rehabilitation setting and mean steps per day (*P=*.34) (Table 2 and Figure 4).

## Discussion

### Principal Results

We found a significant correlation in age in the step activity groups. The patients used the step counter for a mean of 160 (SD 100) days and there was a significantly lower duration of step counter use in the low activity group than in the medium and high activity groups. The overall mean number of daily steps was 5899 (SD 3151), increasing slightly from mean 5191 (SD 3198) steps per day at the first week to mean 7890 (SD 2629) steps per day after 1 year. Nevertheless, this increase might be due to dropout of patients with low activity. Furthermore, the less physically active patients weighed more. There were no significant differences in patients’ treatment or rehabilitation in the three activity groups.

The patients were given the opportunity to wear the Fitbit for up to 365 days after the cardiac event; the mean total days were 160 (SD 100), of which 87% (139/160) were active days. Inactive days might be due to forgetfulness or a decision not to wear the Fitbit every day. A closer look at the data revealed that some patients had 7 to 14 consecutive days without step recordings, which might indicate holiday trips with no Fitbit use. Qualitative studies of the patients’ behavior and motivation in relation to Fitbit wearing are needed to shed light on these issues.

We can compare our results with a study by Izawa et al [18] of cardiac patients who walked a mean of 8609 steps per day 1 month after discharge. In our study, the mean at 30 days was 6362 steps, indicating that our patients were less active at 1 month than those in Izawa et al’s study. The same pattern was observed throughout the year. The mean for the patients in our study was up to 25% fewer steps per day than cardiac patients in comparable studies [15,16,18,21,35,38,39]. The main difference in the studies was the continuity of step counter use. In all studies except ours, the step counter was given to the patient for the first month or for 1 week before the time of measurement (at 1, 3, 6, 9, or 12 months), whereas patients in our study retained the Fitbit for 365 days. This may explain the discrepancy between the studies. There is reason to believe that our findings represent a more realistic picture of walking activity because patients might change their behavior to what is expected on the days of measurement, meaning that they may walk more than usual. This effect might have been eliminated due to the continuous wearing of the Fitbit in our study. None of the previously mentioned studies used the Fitbit Zip, which may prevent adequate comparison of step results. Accuracy studies on Fitbit Zip in healthy adults have revealed satisfactory step measurements in free-living physical activity [40,41], but slow walking speed seems to provide inaccurate step measurements [42]. Older people (>70 years) [43] and heart failure patients [44] walk at a slow speed, which might hamper the Fitbit Zip’s capability to measure steps accurately. Studies of Fitbit Zip’s accuracy when used by cardiac patients are needed.

The explanation for the significant association between long-term use of the Fitbit and high step activity was not identified in this study. In line with other studies [18,21,25,27-31], this might indicate that Fitbit users were encouraged to increase their walking activity. The telerehabilitation setting also provided the patients with goals for daily steps and an opportunity to monitor and follow their own walking activity. In another of Teledi@log’s substudies, Thorup et al [45] found that self-monitoring of steps provided a conscious awareness of walking activity due to the immediate feedback on step activity. In our study, males represented 51 of 64 (80%) participants, almost the same as in comparable studies [12,35,39,46]. The correlation between high BMI and low steps per day has been seen in other studies [13,15,24] and may be considered a health problem for cardiac patients [3]. The significant negative relation between increasing age and steps per day is also evident in other studies [21,25,32,46]. In our study, 22% of the patients were classified as low active (mean <3000 steps/day) and 14% were highly active (walking ≥10,000 steps/day). The remaining 64% were medium active and walked between 3000 and 9999 steps per day. The recommendation of 10,000 daily steps to achieve health benefits appears to be a reasonable estimate of daily activity for healthy adults, but this goal may be too ambitious for people with cardiac disease [17,26,46]. Research suggests that a target of approximately 7000 [35] to 7500 [17] daily steps might reduce waist circumference, BMI, and cardiovascular disease risk factors in patients with coronary artery disease [17,35]. Bearing in mind the dose-response relationship between physical activity and health status [46-48], 7500 daily steps may not be sufficient to reach optimal health status in cardiac patients [17], yet it could be the starting point to improving their physical activity levels. However, experts do not agree on the number of steps needed per day for cardiac patients to provide better health in secondary prevention of cardiac disease. Similar to other studies [13,15,46], our study found no significant association between gender and mean steps per day. Patients who choose cardiac telerehabilitation through the call center did not follow group exercises, whereas patients who chose cardiac rehabilitation at either the health care center or the hospital did follow group exercises. Despite the lack of group exercise, they had the highest mean steps per day (mean 6847, SD 4353) compared to the other cardiac telerehabilitation settings (although the difference was not significant). This might indicate that cardiac telerehabilitation at a call center can support walking activity just as effectively as cardiac telerehabilitation at hospitals and health care centers. In line with this, Thorup et al [45] found that the Fitbit led to self-monitoring, which then led to independence of standardized rehabilitation programs.

### Limitations

Despite the strength of our study, in that the patients’ steps were monitored continuously for 1 year, the results must be viewed cautiously due to the low number of participants. Although Fitbits provide an objective measurement of physical activity, they are not designed to capture different modes of physical activity (eg, cycling and swimming). Furthermore, Fitbits are considered less valid during slow walking and in obese patients [49,50], and there is reason to believe that cardiac patients might have a slower walking pace [24,44]. The Fitbit was also part of a larger telerehabilitation program; therefore, other factors might have influenced the patients’ activity.

### Conclusion

This study has demonstrated that cardiac patients in the Teledi@log program walked a mean of 5899 steps per day in the year after a cardiac event, increasing from a mean 5191 steps per day at the first week to a mean 7890 steps per day after 1 year. In this study, the patients tended to walk less than cardiac patients in comparable studies, but there is reason to believe that this study represents a more realistic picture of walking activity due to the continuation of Fitbit use (mean 160 days). The patients who walked more tended to be of younger age, had a lower BMI, and continued using the Fitbit for a longer period. There were no significant differences in mean steps per day for patients based on their type of treatment or rehabilitation setting. Qualitative studies on the patients’ behavior and motivation regarding Fitbit use are needed to shed light on adherence to and motivation to use the Fitbit.

## Abbreviations

ACS: acute coronary syndrome
BMI: body mass index
CABG: coronary artery bypass graft
PHR: personal health record
